# Characterization of vanillin carbon isotope delta reference materials

**DOI:** 10.1007/s00216-022-04322-x

**Published:** 2022-10-06

**Authors:** Michelle M. G. Chartrand, Juris Meija, Jean-Francois Hélie, Paul Middlestead, Malarvili Ramalingam, Azharuddin Abd Aziz, Zoltan Mester

**Affiliations:** 1 National Research Council Canada, 1200 Montreal Rd., Ottawa, ON K1A 0R6 Canada; 2 Geotop and Département des sciences de la Terre et de l’atmosphère, Université du Québec à Montréal, C.P. 8888 succ. Centre-ville, Montréal, QC H3C 3P8 Canada; 3 Ján Veizer Stable Isotope Laboratory, University of Ottawa Advanced Research Complex, 25 Templeton Street, Ottawa, ON K1N 6N5 Canada; 4 Department of Chemistry Malaysia, Jalan Sultan, 46661 Petaling Jaya, Selangor Malaysia

**Keywords:** Vanillin, Carbon isotope delta measurements, Certified reference material, Food authenticity

## Abstract

Stable carbon isotope ratio measurements are used to investigate the provenance of vanillin. In this study, a variety of commercial vanillin samples and vanilla products were analyzed to provide a frame of reference for the variability of carbon isotope delta values in various vanillin samples, with the results ranging from −20.6 to −36.7‰ relative to the Vienna Peedee Belemnite (VPDB). We present information on the development of two synthetic vanillin reference materials, VANA-1 and VANB-1, prepared in 0.75 g units in glass vials, to be used for the calibration of carbon isotope delta measurements of vanillin and other easily combustible organic materials. Characterization of 40 vials each of VANA-1 and VANB-1 was performed by three laboratories over several measurement sequences. The certified carbon isotope delta values are −31.30 ± 0.06‰ (VANA-1) and −25.85 ± 0.05‰ (VANB-1). These uncertainties, for the 95% confidence level, include considerations for measurement uncertainty, coherence of the reference materials used for calibration, batch homogeneity, and stability during storage and transportation. The results are traceable to the VPDB through a set of nine reference materials (IAEA-CH-6, USGS65, IAEA-600, NBS22, USGS61, IAEA-603, IAEA-610, IAEA-611, and IAEA-612). For up to date certified values, users should refer to doi.org/10.4224/crm.2022.vana-1 and doi.org/10.4224/crm.2022.vanb-1.

**Supplementary Information:**

The online version contains supplementary material available at 10.1007/s00216-022-04322-x.

## Introduction

Determining the authenticity and provenance of food items is an integral part of safeguarding food supply chains [1–3]. Vanilla, a plant which is cultivated in tropical climates, is one of the most widely used food flavors in the world. The main flavor component of vanilla is vanillin, and this ingredient can be found in a wide variety of processed foods [1–4]. Vanillin comprises only a few percent of the vanilla bean, and its extraction is a lengthy and expensive process [3]. The demand for vanillin far outweighs the production of vanilla beans, thus resulting in a 100-fold premium paid for vanillin extracted from vanilla pods compared to synthetic vanillin [1–3]. While most of the global vanillin supply is a synthetic vanillin made from olefin-based precursor guaiacol [3], it is now common to see “biovanillin” made from natural precursors such as rice [4–6].

**Fig. 1:**
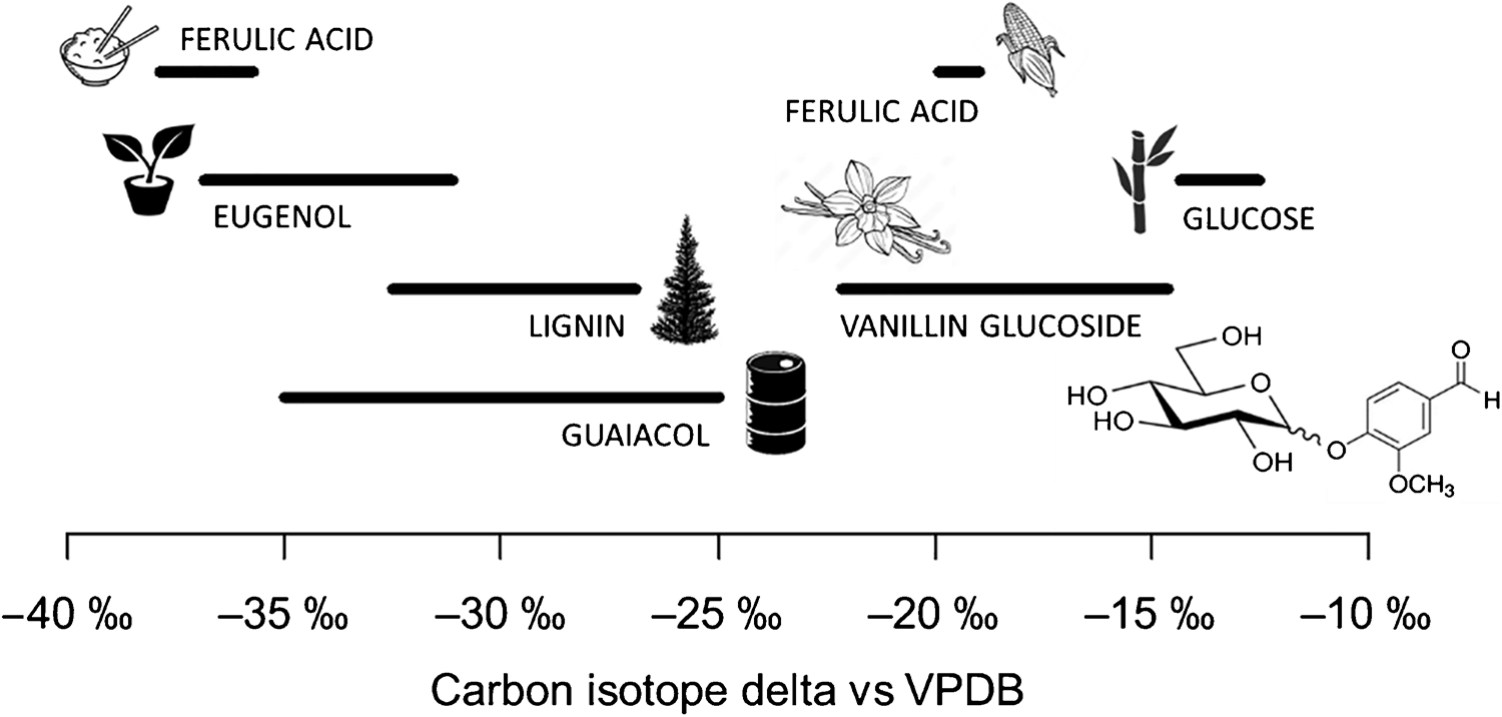
Carbon isotope delta values of vanillin obtained from various sources

Carbon isotope delta, *δ*_VPDB_(^13^C), measurements by isotope ratio mass spectrometry (IRMS) are routinely used to determine the authenticity of vanillin and vanillin-containing food products by determining the source materials from which the vanillin ingredient was derived [2, 6–13]. This technique utilizes the differences between the three main photosynthetic pathways of plants: C3, C4, and crassulacean acid metabolism (CAM) [14–17]. C3 and C4 plants have distinct ranges of carbon isotope delta values, ranging from −35 to −21 ‰ for C3 plants and −16 to −9 ‰ for C4 plants [18]. Vanilla is a CAM plant whose photosynthetic pathways adapt to their growing environmental conditions which results in a wide range of natural variations of its isotope delta values [16]. In addition, there are a number of industrial processes to make food-grade vanillin which leads to a vast range of carbon isotope delta values for vanillin. For example, biosynthesis of vanillin from C4 glucose has the most positive reported carbon isotope delta value, −12.5 ‰, whereas vanillin obtained from rice-derived ferulic acid has the most negative reported carbon isotope delta value, −37.9 ‰ [6]. The *δ*_VPDB_(^13^C) values of vanillin extracted from vanilla pods, ferulic acid derived from corn, guaiacol, lignin, and eugenol are depicted in Fig. 1 [2, 6–13].

To minimize sources of error in carbon isotope delta measurements, the reference materials (RMs) used for calibration to the Vienna Peedee Belemnite (VPDB) should have the same matrix as the sample [19–21]. Several suites of RMs have been recently developed to provide a wider variety of available matrices [22–24]; however, vanillin reference materials are still lacking. This paper describes the measurement of vanillin and vanilla samples to provide a frame of reference for the natural variability of carbon isotope delta values in commercial vanillin. Further, the development of two synthetic vanillin certified reference materials (CRMs), VANA-1 [25] and VANB-1 [26], are described, which are intended for use as a calibrant for carbon isotope delta measurements using IRMS, or as a comprehensive standard for other measurement techniques such as NMR [12, 27].

## Materials and methods

### Vanillin samples

Twenty-two samples of vanillin and vanilla products from various sources were sourced by the National Research Council Canada (NRC) and the Department of Chemistry Malaysia (DoCM) as shown in Table 1. These include a variety of pure vanillin samples from either natural or synthetic sources as well as raw natural materials containing vanillin.

**Table 1:** Additional information for the commercial vanillin and vanilla samples measured for this study

Sample ID	Laboratories providing *δ*(^13^C) measurements	Classification	Details
1	NRC, DoCM, Geotop	Synthetic vanillin	Pure chemical (Alfa Aesar), product of USA
2	NRC, DoCM, UO	Synthetic vanillin	Pure chemical (Fisher Scientific), product of USA
3	NRC, DoCM, Geotop	Synthetic vanillin	Pharmaceutical standard (Sigma Aldrich), product of USA
4	NRC, DoCM, UO	Synthetic vanillin	Pure chemical (Sigma Aldrich), product of China
5	NRC, DoCM, Geotop	Synthetic vanillin	Pure chemical (Fisher Scientific, TCI America)
6	NRC, DoCM, UO	Synthetic vanillin	Pure chemical (Sigma Aldrich)
7	NRC, DoCM, Geotop	Synthetic vanillin	Pure chemical (Sigma Aldrich, Merck), product of France
8	NRC, DoCM, UO	Synthetic vanillin	Pure chemical (Sigma Aldrich), product of China
9	NRC, DoCM	Synthetic vanillin	Pure chemical (Alfa Aesar), product of USA
10	NRC, DoCM, UO	Vanilla powder	Bourbon vanilla powder (Epicureal), imported by Canada
11	NRC, DoCM	Vanilla powder	Bourbon vanilla bean powder (Kiva), product of Madagascar
12	NRC, DoCM, Geotop	Vanilla powder	Bourbon vanilla powder (Organic Traditions), manufactured in Canada
13	NRC	Vanilla extract	Madagascar Bourbon pure vanilla extract, product of USA
14	NRC	Vanilla paste	Bourbon vanilla paste (Epicureal), product of Madagascar and Indonesia
15	NRC	Vanilla extract	Vanilla extract (President’s Choice), product of USA
16	NRC, DoCM	Vanilla beans	Vanilla beans, *V. planifolia* (Stavoren Trading Co.), product of Madagascar
17	NRC, DoCM	Vanillin extracted from lignin	Vanilla powder (Borregard, Sarpsborg, Norway), imported by Malaysia
18	NRC, DoCM	Vanillin extracted from vanilla flavor	Vanilla powder (Kijang), imported by Malaysia
19	NRC, DoCM	Vanillin extracted from vanilla flavor	Vanilla powder (HOI), imported by Malaysia
20	NRC, DoCM	Vanillin extracted from rice ferulic acid	Intercomparison testing platform “Agroisolab-KPT” sample, Agroisolab GmbH, Jülich, Germany
21	NRC, DoCM	Vanillin extracted from vanilla bean	Intercomparison testing platform “Agroisolab-KPT” sample, Agroisolab GmbH, Jülich, Germany
22	NRC, DoCM	Synthetic vanillin	Intercomparison testing platform “Agroisolab-KPT” sample, Agroisolab GmbH, Jülich, Germany

Vanillin extracted from vanilla flavor (samples 18 and 19) were prepared at the DoCM. Thirty grams (30 g) of each vanillin sample powder was weighed into a separate 100 mL beaker and dissolved in 25 mL methanol. The solution was stirred for 10 min prior to being filtered. The filtrate was washed with methanol and dried overnight in a water bath at 80 °C until dryness. The dried crystals were re-dissolved using hot DI water, stirred, and filtered to remove residue and oil. The filtrate was immersed in chilled water to re-form vanillin crystals, which were then filtered, washed with chilled water, and placed in the oven to dry overnight.

For CRM production, two batches of high-purity (> 99%) synthetic vanillin were purchased by the NRC from Canadian chemical suppliers: 1 kg from ACROS Chemicals (Fisher Scientific, Waltham, MA, USA) and 2 kg from Sigma Aldrich (St. Louis, MO, USA). Aliquots from materials were stored in 2 mL glass vials capped with black phenolic caps with a polyethylene conical liner (Wheaton, supplied by Fisher Scientific, Waltham, MA, USA), and kept in a drybox at room temperature until analyzed.

### Carbon isotope delta measurements

Details regarding the instrumentation and procedures used for carbon isotope delta measurements performed at the NRC; the Ján Veizer Stable Isotope Laboratory at the University of Ottawa, Canada (UO); the stable isotope laboratory at the Research Centre in Earth System Dynamics (Geotop) at the Université du Québec à Montréal, Canada; and DoCM are presented in the Electronic supplementary material. Two different sets of calibrators were used to determine carbon isotope delta values relative to the VPDB for vanillin and vanilla samples listed in Table 1: DoCM used BEET-1, FRUT-1, and GALT-1 [23] whereas UO, Geotop, and NRC used IAEA‐CH‐6 [28], USGS65 [29], IAEA-600 [20], NBS22 [28], and USGS61 [30]. Reference materials used for calibration of VANA-1 and VANB-1 are discussed later.

## Results and discussion

### Commercial vanillin sample measurements

**Fig. 2:**
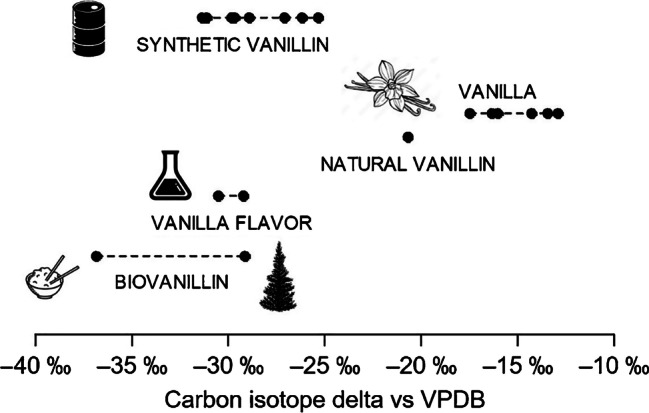
Carbon isotope delta values for samples of vanillin and vanilla samples measured in this study

Carbon isotope delta measurements can be used to differentiate between sources of vanillin [2, 6–13]. In an effort to map the vanillin carbon isotope delta space, the vanilla and vanillin samples described in Table 1 were analyzed (Fig. 2 and Electronic supplementary material Table S1). The agreement in the average reported *δ*_VPDB_(^13^C) values between the laboratories was within 0.4 ‰ for all vanillin samples, and within 1 ‰ for the vanilla samples.

As vanilla undergoes CAM photosynthesis, the carbon isotope delta values can vary from −34 to −10 ‰, depending on the growing conditions [16, 18]. All vanilla samples analyzed for this study exhibited C4-type photosynthesis, indicating a hot and arid climate typical of regions producing vanilla beans. Additionally, while carbon isotope delta measurements can identify natural vanillin, vanillin samples obtained from synthetic or biosynthetic means display largely identical isotopic compositions.

The *δ*_VPDB_(^13^C) of natural vanillin from vanilla beans was −20.6 ‰, consistent with the previously published values for this source of vanillin (−14.6 to −22.2 ‰ [2, 7–13]). The *δ*_VPDB_(^13^C) value of vanillin from rice ferulic acid, −36.8 ‰, also agreed with previous *δ*_VPDB_(^13^C) measurements for vanillin prepared from this natural source (−35.7 to −37.9 ‰ [6, 9, 12, 13]). The vanillin from lignin had a *δ*_VPDB_(^13^C) value of −29.1 ‰, also consistent with reported values (−26.9 to − 32.5‰ [7–10, 12, 13]). The *δ*_VPDB_(^13^C) values of the two vanillin samples from “vanilla flavor,” −29.2 ‰ and −30.5 ‰, were similar to that of the synthetic or biovanillin and agreed well with commercial synthetic vanillin measured in other studies (−28.5 to −32.6 ‰; [10, 11, 13]).

Despite its popularity, the power of carbon isotope delta measurements in provenance studies remains limited. The price of the various precursors of vanillin ranges from a few dollars to thousands of dollars per kilogram. However, the carbon isotope delta values alone are unable to distinguish between the most expensive natural vanillin (1200 USD/kg [3, 4, 6]) and the least expensive biovanillin obtained from cane sugar (0.30 USD/kg [4, 6]). Thus, additional isotopes are often measured or intra-molecular measurements are performed using ^13^C-qNMR [2, 9, 10, 12, 13, 27].

### Development of vanillin CRMs

VANA-1 was prepared from vanillin purchased from ACROS Chemicals, with the appearance of a white powder. The 1 kg bottle was tumbled for 2 h to homogenize the contents, then sieved through a US standard 30 mesh (595 µm) sieve. The sieved VANA-1 was transferred to a 4 L bottle and tumbled for an additional 45 min to further homogenize the sample. VANB-1 was prepared from vanillin purchased from Sigma Aldrich. It appeared to be a mix of white powder and needle-shaped larger crystals. This material was ground in batches using a ball mill with a stainless steel grinding jar and balls and then sieved through a US standard 50 mesh (300 µm) sieve. The sieved VANB-1 was transferred to a 4 L bottle and tumbled for 45 min.

Aliquots of 0.75 g of the prepared VANA-1 and VANB-1 material were transferred into 2 mL glass vials capped with black phenolic caps with a polyethylene conical liner. The vials were sealed in trilaminate bags and stored at room temperature. In total, 1000 units each of VANA-1 and VANB-1 were prepared.

### Stability study

In order to qualify the storage and shipping conditions of a candidate CRM, the stability of the material at various environmental conditions needs to be assessed. Short-term stability studies are intended to model extreme temperature exposures experienced during shipping/transit of the material from the producer to the user laboratories. For each CRM, two unopened vials were stored in the oven (+40 °C) and the freezer (−20 °C). After 16 days, the contents of each vial were measured in triplicate at the NRC using IAEA-CH-6, USGS65, IAEA-600, NBS22, and USGS61 as calibrators (ST1/ST2 vs ST0, Table 2). No effect in the *δ*_VPDB_(^13^C) values was observed.

**Table 2:** Average *δ*_VPDB_(^13^C) values ± 2 SD for VANA-1 and VANB-1 vials stored at various conditions

Material	ST0, ‰	ST1, ‰	ST2, ‰	ST3, ‰	LT1, ‰
VANA‐1	− 31.33 ± 0.06	− 31.36 ± 0.06	− 31.36 ± 0.04	− 31.36 ± 0.04	− 31.32 ± 0.06
VANB-1	− 25.86 ± 0.08	− 25.89 ± 0.04	− 25.86 ± 0.08	− 25.88 ± 0.08	− 25.85 ± 0.06

ST0: closed vials stored at room temperature, analyzed on day 1; ST1: closed vials stored at +40 °C for 16 days; ST2: closed vials stored at −20 °C for 16 days; ST3: open vials stored at room temperature for 16 days; LT1: closed vials stored at room temperature for 23 months in trilaminate bags

In addition to the short-term stability experiment, the impact of loss due to the volatilization of vanillin from the open vials was studied. The caps on two vials each of VANA-1 and VANB-1 were removed; the vials were lightly covered and left open in the fume hood at room temperature. After 16 days, each vial was measured in triplicate (ST3 vs ST0, Table 2). No effect in the *δ*_VPDB_(^13^C) values was observed.

To assess the effect of long-term storage of VANA-1 and VANB-1 at room temperature in trilaminate bags, two unopened units of both VANA-1 and VANB-1 were analyzed 23 months after the initial measurement campaign (LT1 vs ST0, Table 2). No effect in the *δ*_VPDB_(^13^C) values was observed. Thus, both VANA-1 and VANB-1 are deemed stable with respect to long-term storage at room temperature, and no special shipping requirements are warranted.

### Characterization of VANA-1 and VANB-1

Three laboratories — NRC, UO, and Geotop — analyzed a total of 40 vials of VANA-1 and 40 vials of VANB-1 over several measurement sequences, providing a total of nearly 500 measurement results (Electronic supplementary material Table S2). The first set of measurements were performed by all three laboratories using IAEA‐CH‐6, USGS65, IAEA-600, NBS22, and USGS61 as calibrators whose *δ*_VPDB_(^13^C) values are traceable to the VPDB scale defined by both NBS19 and LSVEC (VPDB2006), with *δ*_VPDB_(^13^C, NBS19) =  +1.95 ‰ and *δ*_VPDB_(^13^C, LSVEC) =  −46.6 ‰ [19]. The uncertainties associated with these calibrators [20, 28–30] were enlarged, in quadrature, by 0.029 ‰, to capture the uncertainties due to overall coherence between these calibrators, as described in more detail by Chartrand et al. [23].

**Fig. 3:**
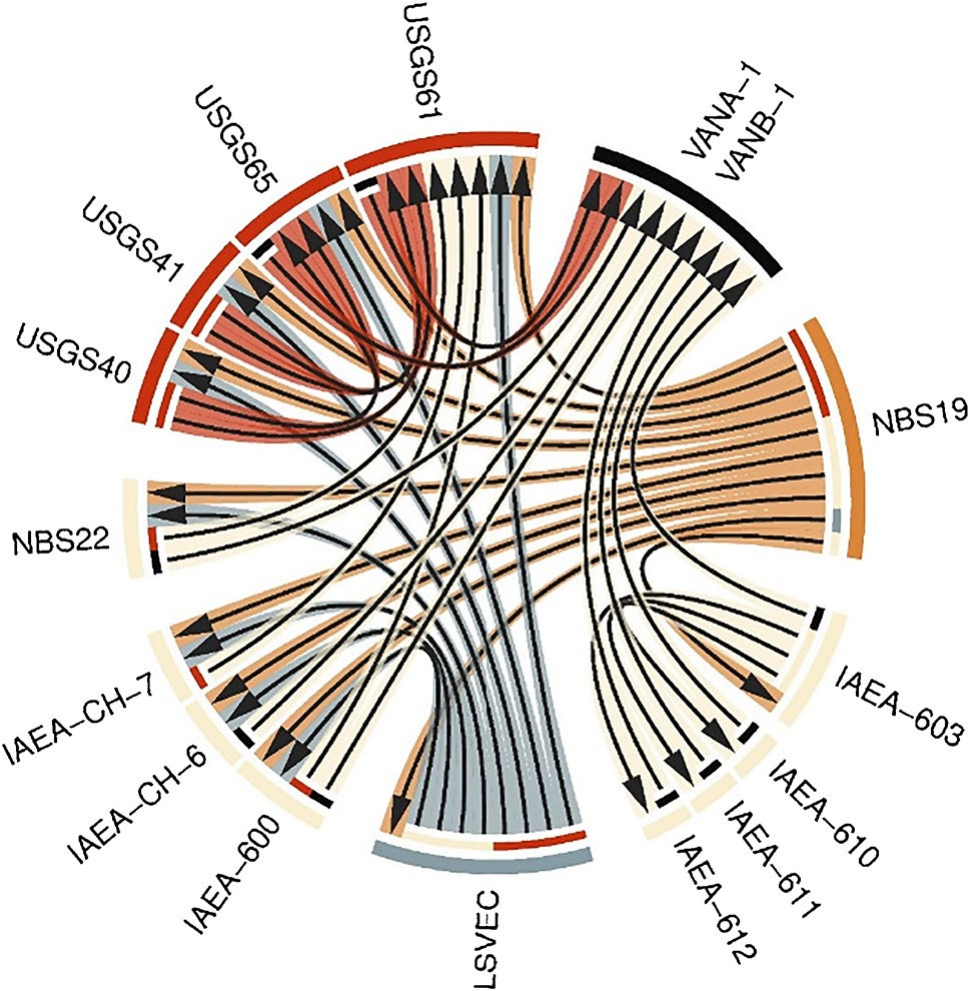
Traceability pathway for carbon isotope delta values in VANA-1 and VANB-1

Additional measurements of VANA-1 and VANB-1 were performed at Geotop and NRC using a set of carbonate standards from the International Atomic Energy Agency, IAEA-603, IAEA-610, IAEA-611, and IAEA-612, whose *δ*(^13^C) values are traceable to the VPDB scale via NBS19 alone (VPDB2020), without relying on the LSVEC as the second scale anchor [31, 32]. Since this realization of the VPDB differs from the conventional NBS19-LSVEC definition, we employed the equation given by Hélie et al. [33] to convert the *δ*(^13^C) values from the VPDB2020 scale to the VPDB2006 scale for these measurements. Table 3 lists the values used for the calibration standards in this work, and Fig. 3 shows the traceability pathways of these materials.

**Table 3:** Re-assigned carbon isotope delta values and the associated standard uncertainties for the reference materials used to calibrate VANA-1 and VANB-1

Reference material	*δ*_VPDB_(^13^C), ‰
IAEA-603	+ 2.474 (*u* = 0.023)
IAEA-610	− 9.145 (*u* = 0.019)
IAEA‐CH‐6	− 10.45 (*u* = 0.049)
USGS65	− 20.29 (*u* = 0.049)
IAEA-600	− 27.77 (*u* = 0.049)
NBS22	− 30.03 (*u* = 0.058)
IAEA-611	− 30.925 (*u* = 0.021)
USGS61	− 35.05 (*u* = 0.049)
IAEA-612	− 36.878 (*u* = 0.026)

### Data reduction and uncertainty evaluation

Data reduction was performed by the NRC. For each measurement sequence (*k* = 1…6) from either of the three laboratories, a linear multi-point calibration curve was established using a laboratory-specific errors-in-variables statistical model. Thus, the individual isotope delta measurements of the calibration standards made by laboratory against the CO_2_ working gas, *d*_*k*_, were modeled as follows:

$${d}_{k,i}\sim normal\left\{{a}_{k}\right\}+{b}_{k}{D}_{i},{u}_{k}$$

where *a*_*k*_ and *b*_*k*_ are the laboratory-specific linear regression parameters (intercept and slope), *u*_*k*_ is the laboratory-specific measurement uncertainty, and *D*_*i*_ is the true isotope delta value associated with each calibrator:

$${D}_{i}\sim normal\left\{{\delta }_{i},u\left({\delta }_{i}\right)\right\}$$

Prior probability distributions for all model parameters were set to be vaguely informative, and the full specification of the statistical model is provided in the Electronic supplementary material.

The vanillin sample measurements were modeled using similar considerations, and the calibrated results were calculated from the appropriate calibration function for each measurement sequence. The obtained carbon isotope delta values for all vanillin samples were then combined with considerations that they arise from a common consensus value affected by random laboratory effect and by the bottle-to-bottle homogeneity. The model fitting was done using Markov Chain Monte Carlo in R using rjags resulting in a single consensus value for carbon isotope delta for each material along with the estimate for the uncertainty due to homogeneity. The results of each individual measurement sequence are shown in the Electronic supplementary material Table S3, and the certified *δ*_VPDB_(^13^C) values for VANA-1 and VANB-1 are shown in Table 4. The standard uncertainty associated with the certified value (*u*) is obtained by combining the uncertainty of the consensus value (*u*_char_) and homogeneity (*u*_hom_). The expanded combined uncertainty (*U*_95% CI_) is taken as *U*_95%_ = 2*u*. As noted before, the uncertainties associated with stability during short- and long-term storage were assessed and considered to be negligible.

**Table 4:** Certified carbon isotope delta values, *δ*_VPDB_(^13^C), for NRC VANA-1 and VANB-1

Material	*δ*_VPDB_(^13^C), ‰	*U*_95% CI_, ‰	*u*, ‰	*u*_char_, ‰	*u*_hom_, ‰
VANA-1	−31.30	0.06	0.03	0.030	0.007
VANB-1	−25.85	0.05	0.03	0.025	0.006

Recent studies [22–24] have provided suites of the same material intended to be used as a set for calibration of samples with similar matrices. In this study, we have provided two CRMs with similar *δ*_VPDB_(^13^C) values, which may be used as a QC material, or as a calibrant for carbon isotope delta measurements. The overall uncertainties associated with the carbon isotope delta values in VANA-1 and VANB-1 are comparable to other organic carbon isotope delta reference materials supplied by USGS, IAEA, and NIST [20]. Carbon isotope delta values can be used to calculate other derived isotopic quantities. We take the isotope ratio value for VPDB as the weighted average from three independent recent studies (Electronic supplementary material, Table S4 [34–36]) devoted to its measurement as *R*(^13^C/^12^C) = 0.011 108(10), and propagate its uncertainty to the derived quantities shown in Table 5 using the NIST Uncertainty Machine [37].

**Table 5:** Derived isotopic quantities for VANA-1 and VANB-1

Material	Isotopic abundance *x*(^13^C), mol/mol	Isotope ratio *R*(^13^C/^12^C), mol/mol	Molar mass *M*(C), g/mol
VANA-1	0.010 646(10)	0.010 760(10)	12.010 682(10)
VANB-1	0.010 705(10)	0.010 821(10)	12.010 741(10)

## Conclusions

To demonstrate the variability of carbon isotope delta measurements in vanillin samples derived from different source materials, we analyzed a variety of commercial vanillin samples and vanilla products, with the results ranging from −20.6 to −36.7 ‰. The results for the vanillin with known provenance were consistent with the previously published values for different vanillin sources. Further, we report the characterization of carbon isotope delta values in two isotope reference materials of synthetic vanillin, VANA-1 and VANB-1, with certified carbon isotope delta values of −31.30 ± 0.06 ‰ and −25.85 ± 0.05 ‰ (*k* = 2) respectively, and traceable to the VPDB through nine reference materials. The 95 % confidence level uncertainties include components for measurement uncertainty, calibration reference material coherence, and bottle-to-bottle homogeneity. The uncertainty due to short-term stability during transport and storage, as well as long-term storage stability, was deemed negligible. VANA-1 and VANB-1 are the first reference materials on track to be characterized not only for bulk carbon isotope delta values, but also for site-specific carbon isotope delta values [27]. VANA-1 and VANB-1 are intended for use as a calibrant or as a QC material for IRMS measurements of vanillin or samples of other similar matrices. A vanillin reference material with a carbon isotope delta value of approximately −15 ‰ might be of future interest.

## Supplementary Information

Below is the link to the electronic supplementary material.

Supplementary file1 (DOCX 37.5 KB)

Supplementary file2 (XLSX 37.5 KB)
