# Supplementary material for: Characterization of vanillin carbon isotope delta reference materials
Source: Anal Bioanal Chem. 2022 Oct 6;414(27):7877–83. doi: 10.1007/s00216-022-04322-x (PMC9568493; doi:10.1007/s00216-022-04322-x)
Supplement: Supplementary file 1 — Supplementary file1 (DOCX 37.5 KB) [file 216_2022_4322_MOESM1_ESM.docx]

**Characterization of Vanillin Carbon Isotope Delta Reference Materials**

**ELECTRONIC SUPPLEMENTARY MATERIAL**

Michelle M.G. Chartrand^1*^, Juris Meija^1^, Jean-Francois Hélie^2^, Paul Middlestead^3^, Malarvili Ramalingam^4^, Azharuddin Abd Aziz^4^, and Zoltan Mester^1^

^1^National Research Council Canada, Metrology, 1200 Montreal Rd., Ottawa, ON, Canada K1A 0R6

^2^Geotop and Département des sciences de la Terre et de l’atmosphère, Université du Québec à Montréal,  C.P. 8888 succ. Centre-ville, Montréal, QC, H3C 3P8, Canada.

^3^Ján Veizer Stable Isotope Laboratory, University of Ottawa Advanced Research Complex, 25 Templeton Street, Ottawa, ON, Canada K1N 6N5

^4^Department of Chemistry Malaysia, Jalan Sultan, 46661 Petaling Jaya, Selangor, Malaysia

*Instruments and procedures for δ_VPDB_(^13^C) measurements at NRC, UO, Geotop and DoCM*

Isotope delta measurements of carbon in a sample are expressed on the VPDB scale:

*δ*_VPDB_(^13^C) = *R*_sample_(^13^C/^12^C)/R_VPDB_(^13^C/^12^C) – 1 (1)

where *R*_sample_ is the carbon isotope ratio of the sample, and VPDB denotes Vienna Peedee Belemnite. Since 2006, the VPDB carbon isotope delta scale is realized using two modern CRMs, NBS19 and LSVEC, whose *δ*_VPDB_(^13^C) values are set to +1.95 ‰ and –46.6 ‰ against VPDB, respectively [1].

The NRC analyzed all commercial vanillin and vanilla samples for *δ*_VPDB_(^13^C) measurements, the Department of Chemistry Malaysia (DoCM) analyzed 19 of the 22 vanilla and vanillin samples, and both the Ján Veizer Stable Isotope Laboratory (UO) at the University of Ottawa, Canada, and the stable isotope laboratory at the Research Centre in Earth System Dynamics (Geotop) at the Université du Québec à Montréal, Canada each measured five of these samples (Table 1).

The initial screening of the three CRM candidate materials was performed at the NRC, and two candidate materials, VANA-1 and VANB-1, were chosen for CRM development. The NRC conducted a stability assessment of VANA-1 and VANB-1, and combined all analysis sequences performed at the NRC, UO and Geotop to assign the *δ*_VPDB_(^13^C) value and associated uncertainty for these materials.

All *δ*_VPDB_(^13^C) measurements performed at NRC followed a standard procedure for carbon isotope delta analysis, and are described in detail elsewhere [2]. RMs and samples and RMs were weighed into 5×3.5 mm tin capsules (Elemental Microanalysis; Okehampton, UK). The mass of vanillin analyzed was ~ 650 µg, and the mass of RMs weighed was based on the carbon composition of the RM to achieve approximately the same total amount of carbon content for all measurements. Carbon isotope delta measurements were carried out on an elemental analyzer (EA, Vario EL III, Elementar Americas Inc., Mt. Laurel, NJ, USA or a Costech ECS4010,Costech Anlaytical Inc,Valencia, CA,USA) using a Conflow III (Thermo Fisher; Bremen, Germany) interfaced with a Delta^+^XP isotope ratio mass spectrometer (Thermo Fisher; Bremen, Germany). The combustion reactor was held at 980 ^o^C (Costech) and 950 ^o^C (Vario), the reduction reactor was held at 650 ^o^C (Costech) and 500 ^o^C (Vario). Both the combustion and reduction reactors were packed according to the manufacturers’ specifications for CN measurements. There was negligible difference in *δ*_VPDB_(^13^C) values between the two EAs. The helium carrier gas (99.999 %; Air Liquide; Montreal, QC, Canada) flow rate was measured at ~100 mL/min (Costech) and ~150 mL/min (Vario). Helium dilution on the Conflow III was 0.5 bar (7 psi) for all analyses.

The DoCM weighed ~ 800 µg of vanillin into 6×4 mm tin capsules (Sercon), and analyzed on a Europa EA-GSL Sample preparation system, interfaced to a Sercon 20-22 isotope ratio mass spectrometer (Sercon Ltd., Crewe, UK) running in continuous flow mode. On the EA, the combustion and reduction reactors were set to 1030 ^o^C and 600 ^o^C, respectively, and the helium carrier gas (99.999 %; (Air Liquide, Malaysia) flow rate was 52 mL/min.

UO measured ~ 500 µg of samples and appropriate masses of RMs into 5×3.5 mm tin capsules (Elemental Microanalysis; Okehampton, UK), and analyzed using a Vario Isotope Cube EA (Elementar Langenselbold, Germany), interfaced to a Delta Advantage isotope ratio mass spectrometer (Thermo Fisher; Bremen, Germany) via a Conflow IV interface (Thermo Fisher; Bremen, Germany). On the EA, the combustion and reduction reactors were held at 950 ^o^C and 600 ^o^C, respectively, and the helium carrier gas (99.999 %; Messer) flow rate was 230 mL/min. Helium dilution on the Conflow IV was set to 50 % for analyses.

Geotop weighed ~ 800 µg of vanillin samples and appropriate masses of RMs into 8×5 mm tin capsules (Elemental Microanalysis; Okehampton, UK), and were measured on a Vario Micro Cube EA (Elementar Langenselbold, Germany), interfaced to an IsoPrime 100 (Micromass, now Elementar, Cheadle, UK)) in continuous flow mode. The EA combustion and reduction reactors were set to 1040 ^o^C and 850 ^o^C, respectively, with a helium (99.999 %; Praxair, now Linde) flow rate of 200 ml/min. Helium dilution on the Micromass diluter was set to dilute the CO_2_ signal 35-fold for the analysis by adjusting the Nupro metering valve accordingly.

All VANA-1 and VANB-1 analysis sequences performed at NRC, DoCM, UO and Geotop included blank capsules to evaluate for blank corrections, several sets of RMs, and QC samples that were interspersed throughout the sequence at regular intervals. The NRC also included an additional RM sample, IAEA-600, throughout the sequence to account for instrument drift. Prior to analysis, all laboratories performed a daily start up procedure, and for all analysis sequences across all laboratories, the measured parameters were deemed acceptable.

For the ^17^O correction, the NRC and DoCM used their respective internal spreadsheet to apply the recommended IUPAC linear approximation algorithm [3], UO used the Santrock, Studley and Hayes (SSH) algorithm [4] in the IsoDat software, and Geotop used the SSH correction method in the IonVantage software, but updated the *R*(^13^C/^12^C), *R*(^17^O/^16^O) and *R*(^18^O/^16^O) values for the VPDB to those recommended by IUPAC [3]. All laboratories evaluated for blank and drift; Geotop applied a blank correction, and the NRC applied a drift correction to one of the two VANA-1 characterization sequences, and to both VANB-1 characterization sequences. Each laboratory also used their own internal QC materials, and all average QC results for each laboratory were within 0.1 ‰ of their accepted *δ*_VPDB_(^13^C) values.

*Specifications of the statistical model*

Carbon isotope delta measurements were modelled statistically using linear multi-point calibration curve with laboratory-specific errors-in-variables statistical model and allowance for two random effects: a between-unit homogeneity effect and the random laboratory effect. Model fitting was done with Markov Chain Monte Carlo in R using rjags package. The relevant model code is shown below.

mod_str = "

model{

##############################################################################

# Calibration curves for each laboratory

##############################################################################

for(i in 1:N_labs) {

# Non-informative priors for the intercepts and slopes

intercept[i] ~ dnorm(0, 1.0/50^2)

slope[i] ~ dnorm(1, 1.0/0.10^2)

}

# calibration standards

for(i in 1:length(cal_x)){

# Prior for cal_x_true (+/- 0.10 permilles from the actual values)

cal_x_true[i] ~ dnorm(priors_cal_x_true[i], 1.0/0.10^2)

# Likelihood for x

cal_x[i] ~ dnorm( cal_x_true[i], 1.0/cal_u2x[i] )

}

# Half-Cauchy prior for the measurement uncertainty for each lab

for(i in 1:N_labs){ cal_uy[i] ~ dt(0, 1.0/0.10^2, 1)T(0,) }

# likelihood for the measured isotope deltas

for(i in 1:length(cal_y)){

# Regression model

cal_y_true[i] <- intercept[cal_lab[i]] + slope[cal_lab[i]] * cal_x_true[cal_y_id[i]]

cal_y[i] ~ dnorm(cal_y_true[i], 1.0/cal_uy[cal_lab[i]]^2)

}

##############################################################################

# Measurements of the sample

##############################################################################

# Likelihood

for ( i in 1:N_samples_measurements ) {

samples_y[i] ~ dnorm( samples_y_pred[i] , 1.0/sigma[samples_lab[i]]^2 )

samples_x_pred[i] <- mu + e_lab[samples_lab[i]] + e_hom[samples_unit[i]]

samples_y_pred[i] <- intercept[samples_lab[i]] + slope[samples_lab[i]] * samples_x_pred[i]

}

for ( i in 1:N_labs ) { sigma[i] ~ dt(0,1.0/0.10^2 , 1)T(0,) }

# Grand mean

mu ~ dnorm(-20,1.0/60^2) # weak prior

# Laboratory effect

for ( i in 1:N_labs ) { e_lab[i] ~ dnorm( 0.0 , 1.0/lab_SD^2 ) }

lab_SD ~ dt( 0 , 1.0/1.0^2 , 1 )T(0,) # half-Cauchy prior

# Homogeneity effect

for ( i in 1:N_samples_units ) { e_hom[i] ~ dnorm( 0.0 , 1.0/hom_SD^2 ) }

hom_SD ~ dt( 0 , 1.0/0.05^2 , 1 )T(0,) # half-Cauchy prior

# Individual laboratory results

for ( i in 1:N_labs ){ results_lab[i] <- mu + e_lab[i] }

}

"

setwd({SET FILEPATH})

require(readxl)

require(rjags)

require(lme4)

reduce_data = function(material){

d0 = data.frame(read_xlsx('VAN SI_FINAL.xlsx', sheet = 'Table S2', skip=3))

d0 = data.frame(d0[-1,])

# keep only the data for the selected material

id_sample = which(d0[,'H'] != material)

d0[id_sample, c('G','I','J')] <- NA

id_lab = unique(na.omit(d0[,'G']))

d0[!(d0[,'D'] %in% id_lab),c('D','E','F')] <- NA

# crm & lab & sample ids

cal_names = levels(factor(c(na.omit(d0[,'A']))))

cal_ids = c(factor(cal_names))

lab_names = levels(factor(c(na.omit(d0[,'D']))))

lab_ids = c(factor(lab_names))

unit_nrs = levels(factor(c(na.omit(d0[,'I']))))

unit_ids = c(factor(unit_nrs))

o = order(cal_ids[match(c(na.omit(d0[,'A'])), cal_names)])

df = list(cal_x = as.double(c(na.omit(d0[,'B']))[o]),

cal_u2x = as.double(c(na.omit(d0[,'C']))[o])^2,

cal_y = as.double(c(na.omit(d0[,'F']))),

cal_y_id = cal_ids[match(c(na.omit(d0[,'E'])), cal_names)],

cal_lab = lab_ids[match(c(na.omit(d0[,'D'])), lab_names)],

samples_y = as.double(c(na.omit(d0[,'J']))),

samples_lab = match(c(na.omit(d0[,'G'])), lab_names),

samples_unit = unit_ids[match(c(na.omit(d0[,'I'])), unit_nrs)],

N_labs = length(lab_ids),

N_samples_measurements = length(c(na.omit(d0[,'J']))),

N_samples_units = length(unique(c(na.omit(d0[,'I'])))),

priors_cal_x_true = as.double(c(na.omit(d0[,'B']))[o])

)

# OLS-based initial values of the regression coefficients

init.fits <- coef(lmList(cal_y ~ cal_x | cal_lab, data.frame(cal_x = df$cal_x[df$cal_y_id], cal_y = df$cal_y, cal_lab = df$cal_lab)))

samples_x = (df$samples_y - init.fits[df$samples_lab,1])/init.fits[df$samples_lab,2]

jags_inits = function() list("intercept" = init.fits[,1], "slope" = init.fits[,2],

"cal_x_true" = df$cal_x, "mu" = mean(samples_x),

"e_lab" = aggregate(samples_x, list(df$samples_lab), mean)[,2] - mean(samples_x),

"e_hom" = aggregate(samples_x, list(df$samples_unit), mean)[,2] - mean(samples_x),

"lab_SD" = 0.05, "hom_SD" = 0.05, "sigma" = rep(0.05, df$N_labs)

)

## Compile the JAGS model

N.mcmc = 1e5

jmod = jags.model(file = textConnection(mod_str), data = df, inits = jags_inits, n.adapt = N.mcmc, n.chains=10, quiet=FALSE)

## Sample from the posterior

samples = coda.samples(jmod, inits = jags_inits,

c("mu","lab_SD","hom_SD","results_lab"),

n.iter = N.mcmc, thin = 10)

jags_s = summary(samples)

output = jags_s$statistics[,c('Mean','SD')]

rownames(output)[grepl('results_lab',rownames(output))] = lab_names[lab_ids]

return(output)

}

# Perform data analysis for VANA-1 and write results to a CSV file

material='VANA-1'

d=reduce_data(material)

write.csv(d, paste0(material, '_', tolower(format(Sys.time(), "%d%b%Y")), '.csv'))

# Perform data analysis for VANB-1 and write results to a CSV file

material='VANB-1'

d=reduce_data(material)

write.csv(d, paste0(material, '_', tolower(format(Sys.time(), "%d%b%Y")), '.csv'))

# END

**REFERENCES**

1. Coplen TB, Brand WA, Gehre M, Gröning M, Meijer HAJ, Toman B, et al. New Guidelines for δ13C Measurements. Anal Chem. 2006;78(7):2439-2441. DOI: 10.1021/ac052027c

2. Chartrand MMG, Meija J, Kumkrong P, Mester Z. Three certified sugar reference materials for carbon isotope delta measurements. Rapid Comm Mass Spectrom. 2019;33(3):272-280. DOI: 10.1002/rcm.8357

3. Brand WA, Assonov SS, Coplen TB. Correction for the 17O interference in δ(13C) measurements when analyzing CO2 with stable isotope mass spectrometry (IUPAC Technical Report). Pure Appl Chem. 2010;82:1719-1733. DOI: 10.1351/PAC-REP-09-01-05

4. Santrock J, Studley SA, Hayes JM. Isotopic Analyses Based on the Mass Spectrum of Carbon Dioxide. Anal Chem. 1985;57:1444-1448.
